# Using Harmony Search Algorithm in Neural Networks to Improve Fraud Detection in Banking System

**DOI:** 10.1155/2020/6503459

**Published:** 2020-02-08

**Authors:** Sajjad Daliri

**Affiliations:** Department of Computer Engineering, Science and Research Branch, Islamic Azad University, Tehran, Iran

## Abstract

Financial fraud is among the main problems undermining the confidence of customers in addition to incurring economic losses to banks and financial institutions. In recent years, along with the proliferation of fraud, financial institutions began looking for ways to find a suitable solution in the fight against fraud. Given the advanced and varied changes in methods of fraud, extensive research has been conducted to detect fraud. In this paper, the Artificial Neural Network technique and Harmony Search Algorithm are used to detect fraud. In the proposed method, hidden patterns between normal and fraudulent customers' information are searched. Given that fraudulent behavior could be detected and stopped before they take place, the results of the proposed system show that it has an acceptable capability in fraud detection.

## 1. Introduction

Fraud in the financial system is known as abuse of the system to improve profitability of a person or an organization. In a competition-based environment, the occurrence of fraud can cause a critical problem in business. This issue has become critically serious in recent years [1]. Recently, fraud has been dramatically increasing incurring billion-dollar annual losses to the owners of financial institutions and banks. On the other hand, the advancement of technology in various fields has caused generation of data in high volumes. The volume of data is positively and directly related to the complication of interrelationships [2]. With regard to the issue, data mining is used as exploratory data analysis with the aid of other sciences in which exploration of hidden and unknown information out of the bulk of data is under focus. Access to hidden information contained in big data is more difficult and more complicated. The science of data mining with other methods such as machine learning, databases, and artificial intelligence has spread to detect patterns among such data [3, 4].

## 2. Review of the Related Literature

Since the advent of business activities, some individuals aimed to increase their own profits and incur losses to companies and products of others. In the past, no considerable financial exchanges occurred; however, gradually, the increase in the population and closer communications amplified financial exchanges, and at the same time fraud has also increased [5]. Nevertheless, today, given the new technologies, costs related to fraud have fallen dramatically, yet fraud methods have also progressed. Recently, the issues of telecommunications, e-commerce, and provision of new services have led to the occurrence of fraud in new ways with different dimensions. With the increasing use of the Internet, new types of fraud have appeared, but with the rise of security systems to prevent fraud, there has not been significant progress due to lack of appropriate patterns [6].

Frauds such as technical fraud that exploit weaknesses in the system mostly occur when a new system is introduced, and developers are unaware of those weaknesses. Fraud often occurs when there is unauthorized access to customer accounts. A subscription fraud occurs when users subscribe to a system without any intention to use it. In this case, other people may use their unused subscriptions [7]. However, much of what happens as fraudulence is the use of social engineering. In this case, swindlers use their skills to obtain detailed information about the system and use it instead of trying to discover the weaknesses of a financial system [8].

In a research study [9], a two-layer system was proposed. In the first layer, general rules and specific rules of each customer are located. The degree of suspiciousness of a transaction can be signified in this layer. In the second layer, techniques of Game theory are used to detect fraud. In another research study [10], a neural network was used to detect fraud. For modeling the neural network, a nonlinear discriminant analysis was used. Moreover, to reduce the volume of calculations, a scoring system was used for transactions. The results revealed that the scoring system had provided better results and higher performance compared with the method in which all transactions are evaluated. However, in another research [11], researchers concluded that, when the financial ratio is used as data, using a neural network has premier performance than other methods. In this research, the dataset of 54 firms, each with 20 attributes were used.

In a study [12], Bank Sealer was proposed. Bank Sealer is a decision support system for online banking fraud analysis and investigation. In the first step, the system quantifies the anomaly of a transaction based on customer historical data. In the second step, it finds the cluster wherein users with similar behavior are included, and finally with the help of information obtained, anomalous or normal transaction is proved.

There are methods grounded on the genetic algorithm (GA) such as [9], where a combination of a GA and a Support Vector Machine (SVM) is presented to detect anomalies in data. In the proposed method, a GA has been used to select the best subset of attributes showing anomalies in the system. Then, the dataset has been applied to SVM for training. However, there are methods based on data mining techniques such as [13], which use a distributed approach for fraud detection. In the study, a large dataset of labeled data with algorithms such as C4.5, ID3, Ripper, and CART has been used.

## 3. The Proposed Method

In the present research study, a hybrid system based on the Artificial Neural Network (ANN) technique and Harmony Search algorithm (HAA) is used to detect fraud. The system uses HSA for optimizing the parameters of ANN, while ANN itself is used for fraud detection.

**Figure 1:**
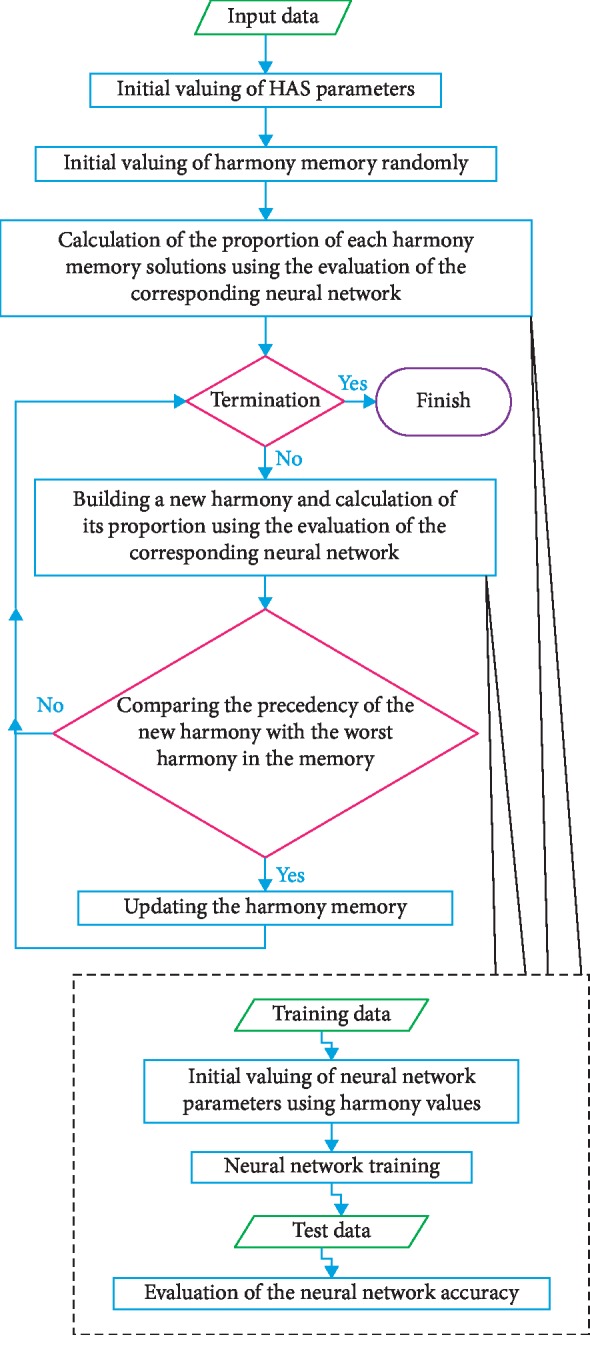
Flowchart of NNHS.

The proposed system is shown as a flowchart in Figure 1. The figure illustrates how ANN is integrated into HSA. Obviously, to calculate the proportion of the solutions, HAS requires building a corresponding ANN. Finally, after building and training of the ANN neural network corresponding to each solution, the performance of ANN is recorded as the proportion of HSA solutions. The method proposed in this study is termed NNHS.

In the neural network used in the present research, back propagation is used for learning, so that the network learns the patterns between inputs and outputs. In this way, for each case in the training data, the desired output is prerecorded.

**Figure 2:**
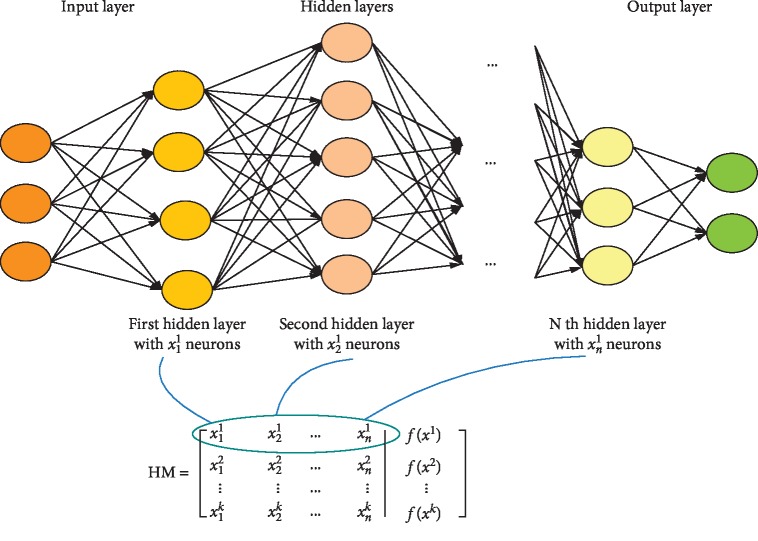
Determining the parameters of the neural network using HSA.

This system continues, as long as the difference between the network outputs and the data outputs is as low as possible. In this network, weights are randomly selected, and then based on how much difference exists between the output from the network and the output recorded in the dataset, weights will be adjusted at each stage. As expressed, HAS is used to determine the parameters of ANN. The structure of the neural network using the harmony memory can be seen in Figure 2.

The HSA task is to find the most suitable structure for the neural network. At the beginning of the execution of the algorithm, parameters such as the size of a harmony memory, the rate of consideration of a harmony memory, the adjustment rate of pitch, and other values are set. The next step is to create the first-generation algorithm randomly. After this step (after each iteration), new harmonies are generated, and accordingly via building, the corresponding neural network is calculated and accuracy is determined. The next step is to update the memory where if the proportion of the New Harmony is lower than the worst proportion recorded in the system, the new one replaces the worst and thus the harmony memory will be updated. In the end, the algorithm stops after performing the specified number of iterations, which is the condition for the termination of the proposed algorithm, and the best harmonies recorded are extracted as the most appropriate solution.

### 3.1. Research Data

The dataset of the present research study is the German Dataset used to evaluate and test the proposed system. This dataset is available at the UCI website and is used in many studies [14]. This dataset helps provide a better comparison with other methods. It contains 100 records of which 300 cases are abnormal and 700 cases are normal.

### 3.2. Test Data

**Figure 3:**
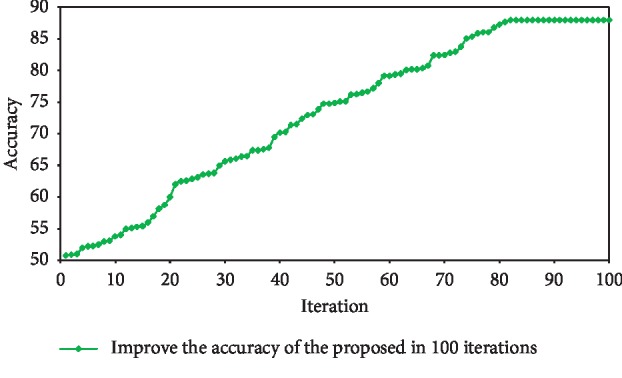
The accuracy of NNHS in different generations.

To improve the testing accurateness, accuracy was used. With an initial population of 150, HAS was executed over 100 iterations. To obtain more reliable results than the proposed system, the test was executed over 10 iterations with the same conditions, and the results were averaged. In Figure 3, accuracy is displayed in 100 generations.

**Figure 4:**
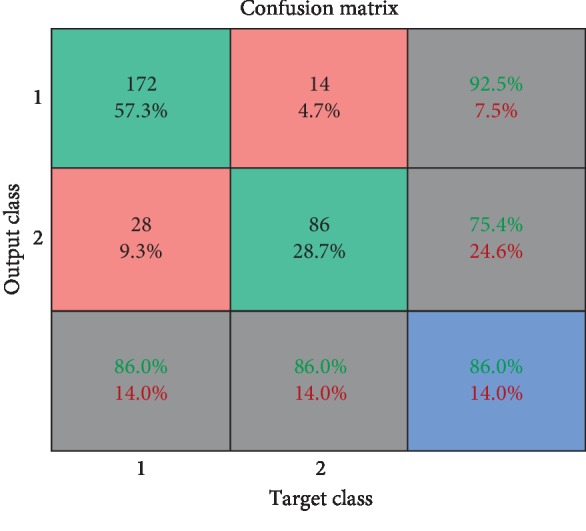
The correlation matrix of our proposed method, NNHS.

As seen in the Figure 3, after 81 generations, accuracy reaches 88%. In this study, as mentioned earlier, after 10 iterations, the test result averages 86% of accuracy. In the second phase of the test, the scattering matrix (SM) was used. The SM calculation method is given in Table 1, and the numerical comparison progress is shown in Figure 4.

## 4. Comparison of the Results

First, NNHS is compared with other types of methods. Hayashi et al. evaluated the criteria of accuracy. They used of the recursive-rule extraction algorithm to detect fraud. In Table 2, NNHS is compared with the method of Hayashi et al. [15].

In another study conducted by Hassan et al., the recursive value was evaluated. The results of this method are also compared with NNHS in Table 3 [16].

Considering other methods, one of the main advantages of NNHS is the improvement of the parameters of ANN using HSA. However, according to the structure of the proposed network, the method is troubled against data with unbalanced information, and detection of the hidden pattern in this type of data has been exposed to many problems.

## 5. Conclusion

In the present study, a fraud-detection model termed NNHS was proposed through the integration of ANN and HSA. The proposed system offers a solution based on HAS succeeding in predicting the best structure for ANN and detecting the hidden algorithm in the mass of data. The results of the comparisons have shown that the best accuracy obtained from the German dataset for the proposed system is 86. In addition, the best value obtained for the same recall criteria is 87. However, the values obtained in [15] and [16] were, respectively, 81.53 and 76.8. Therefore, the results of the evaluation show that having a relatively high performance, NNHS has been able to detect dishonest customers with comparatively high accuracy.

## Figures and Tables

**Table 1:** The SM calculation process.

TN	FN	Negative predictive value = TN/TN + FN
		False omission rate = FN/TN + FN

FP	TP	Precision = TP/TP + FP
		False discovery rate = FP/TP + FP

True negative rate = TN/TN + FP	True positive rate = TP/TP + FN	Accuracy = (TP + TN)/total
False positive rate = FP/TN + FP	False negative rate = FN/TP + FN	Misclassification rate = (FP + FN)/TN + FN + T\ + FP

**Table 2:** Comparison of results of [15].

Method	Training data	Test data	Training accuracy	Test accuracy
Hayashi et al. [15]	700	300	81.32 ± 1.02	80.53 ± 0.88
NNHS	700	300	88	86

**Table 3:** Comparison of results of NNHS and Hassan et al. [16].

Method	Training data	Test data	Recall
Hassan et al. [16]	700	300	76.8
NNHS	700	300	87

## Data Availability

No data were used to support this study.
